# Molecular Identification and Phylogenetic Analysis of *Heterakis dispar* Isolated from Geese

**DOI:** 10.2478/s11686-019-00112-1

**Published:** 2019-09-11

**Authors:** Kamila Bobrek, Joanna Hildebrand, Joanna Urbanowicz, Andrzej Gaweł

**Affiliations:** 1 Department of Epizootiology with Clinic of Birds and Exotic Animals, Faculty of Veterinary Medicine, Wrocław University of Environmental and Life Sciences, pl. Grunwaldzki 45, 50-366 Wrocław, Poland; 2 Department of Parasitology, Institute of Genetics and Microbiology, University of Wrocław, Przybyszewskiego 63/77, 51-148 Wrocław, Poland

**Keywords:** *Heterakis dispar*, Parasite, 18S rRNA, ITS1-5.8rRNA-ITS2, Geese

## Abstract

**Purpose:**

Heterakidosis is a common parasitic infection caused in domestic birds by* Heterakis* species:* Heterakis gallinarum*,* H. isolonche*, and* H. dispar*. Among them, the best described species is* H. gallinarum*, noted mainly in gallinaceous birds. In waterfowl,* H. dispar* is the predominant species. The variations in morphology and host specificity qualify* H. dispar* as a different species, but the phylogenetic relationships between heterakids were unclear for a long time, because of a lack of* H. dispar* sequences.

**Methods:**

The authors provided the molecular data for* H. dispar* and analyzed the obtained sequences of the partial 18S rRNA gene and region ITS1-5.8SrRNA-ITS2 with the homological sequences.

**Results:**

The 18S rRNA PCR product of* H. dispar *was about 800 bp, and the ITS-5.8S-ITS2 PCR product was about 920 bp, noticeably smaller size compared to* H. gallinarum* product. The BLAST analysis of* H. dispar* 18S sequence showed a 99% similarity with the sequences of* Heterakis gallinarum* and* Ascaridia galli*, 98% with* A. nymphii*, but only 94% with the sequence of* Heterakis* sp. Our ITS sequence of* H. dispar* was almost identical to the* H. isolonche* isolate, there is only one nucleotide of difference among the 943 sites analyzed. It also showed a lower similarity to the ITS sequences of* H. gallinarum* (88%),* H. spumosa* (87%), and* H. dahomensis* (87%).

**Conclusions:**

In our phylogenetic analysis, it is the first attempt at the reconstruction of relationships within this superfamily Heterakoidea based on 18S rDNA and ITS region.

## Introduction

Heterakidosis is a common parasitic infection in birds but rarely in rodents. It is caused by the *Heterakis* species (Nematoda, Secernentea, Ascaridida, Heterakoidea, and Heterakoidea). These nematodes infect the ceca of numerous species of wild and domestic birds [1, 2]. In domestic birds, three species of *Heterakis,* differentiated mostly by the morphological characters of males are noted and described: *Heterakis gallinarum* (Schrank, 1788) noted mainly in gallinaceous birds such as chicken, turkey, guinea fowl, partridge, quail, but also in waterfowl. *Heterakis isolonche* (Linstow, 1906) is common in pheasants, but has also been recovered from ducks, turkey, grouse, prairie chicken, and quail, and *Heterakis dispar* (Schrank, 1790), reported in geese and ducks [3–8]. The life cycle of heterakids is direct: eggs are passed in feces and embryonate in the environment within 2 weeks, the infective eggs are ingested by the host directly or birds can be infected by eating earthworms, which are indicated as a paratenic host or mechanical transport host [9, 10]. The infection occurs mostly in poultry kept on litter or that has come in contact with soil in pastures [11, 12].

Among bird’s *Heterakis* species, the best described is *H. gallinarum*. This species is able to infect waterfowl and gallinaceous birds and causes an inflammation of ceca resulting in wall thickness. *H. gallinarum* might also be a vector of *Histomonas meleagridis*, a protozoan which is an etiological agent of histomoniasis—a fatal disease in turkeys and hens. The body length of an *H. gallinarum* female is 10–15 mm approximately and the male 7–13 mm. The male has 12 pairs of tail papillae and its spicules are of various lengths—the left spicule is 3.5 times longer than the right one [5, 13].

*Heterakis isolonche* is similar in morphology to *H. gallinarum* (females measure 9–12 mm and males 6–15 mm), but the spicules of males are long and of equal length. The species invasions are connected with high mortality, especially in pheasants in which the nodular lesions are observed [14].

*Heterakis dispar* is the biggest species of bird’s heterakids (female 16–23 mm, male 7–18 mm), but it is considered less pathogenic than *H. gallinarum* and *H. isolonche.* The characteristic features of the male are 13 pairs of tail papillae and short, equal-length spicules.

Not much information is available about *H. dispar* including epizootiology and pathogenicity [1, 5, 13]. Because of differences in morphology and host specificity *H. dispar* was qualified as a different species than *H. gallinarum* and *H. isolonche,* but the phylogenetic relationships were unclear for a long time, because of a lack of *H*. *dispar* sequences.

The authors provided the molecular data for *H. dispar* and analyzed the obtained sequences of the partial 18S rRNA gene and region ITS1-5.8SrRNA-ITS2 with the homological sequences available in the GenBank database, to complete the phylogenetic relationship within the *Heterakis* genus.

## Materials and Methods

### Necropsy and Parasites

Adult nematodes were collected from the ceca of naturally infected geese which were delivered to the Department of Epizootiology and Clinic of Birds and Exotic Animals, Faculty of Veterinary Medicine in Wrocław for a necropsy and diagnostic analysis. Of the four geese, three of them were infected with *Heterakis* sp.

The nematodes were washed in physiological saline, counted, and identified preliminarily by sex and species based on the morphological characters of males including the size of the parasite, the length of the spicules and the number of caudal papillae [5, 9]. For the DNA extraction, five randomly selected parasites from each goose were chosen.

### DNA Extraction and PCR Reactions

The DNA was extracted using a GeneMATRIX Tissue DNA Purification Kit (EURx, Gdansk, Poland) in accordance with the manufacturer’s instructions and stored at – 20 °C until used.

For 18S rRNA and ITS-5.8rRNA-ITS2 region, PCR reactions were performed using a 12.5 μl 2xPCR Master Mix Plus (A&A Biotechnology, Gdynia, Poland); 0.2 μl of each primer, i.e., Nem 18S-F1 with Nem 18S-R1 for 18S rRNA amplification and Primer2 forward with Primer2 reverse for ITS-5.8rRNA-ITS2 fragment [15, 16]; 2 μl DNA and RNAse free water (A&A Biotechnology, Gdynia, Poland) for a total volume 25 μl.

The PCR conditions for the amplification of 18S rRNA were as follows: initial denaturation at 94 °C for 3 min, 35 cycles of denaturation at 90 °C for 30 s, primer annealing at 55 °C for 1 min, and extension at 72 °C for 1.5 min. The final extension was at 72 °C for 10 min [17]. The PCR cycling parameters for the ITS-5.8rRNA-ITS2 amplification consisted of an initial denaturation at 95 °C for 10 min, followed by 35 cycles of denaturation at 95 °C for 30 s, primer annealing at 67.6 °C for 1 min, and extension at 72 °C for 1 min. The final extension was at 72 °C for 10 min. [16].

PCR products from both reactions were visualized with 2% agarose gel containing a Midori Green advance DNA strain (NIPPON Genetics Europe GmbH, Dueren, Germany) under UV light. Positive products were isolated from agarose gel using a Gel Out Concentrator Kit (A&A Biotechnology, Gdynia, Poland) and were subsequently sent to Macrogen (Amsterdam, Netherlands) for sequencing.

### Sequencing and Phylogenetic Analysis

The nucleotide sequences obtained in this study were edited; chromatograms were inspected visually and then aligned with similar sequences available in the National Center for Biotechnology Information (NCBI) GenBank database using CLUSTALW in MEGA7 package [18]. Identical 18S rRNA and ITS region sequences were observed from all nematode specimens. The received sequences of *H. dispar* have been deposited in the EMBL database under accession numbers MG763171 (18S rRNA) and MF319969 (ITS1-5.8SrRNA-ITS2).

Phylogenetic analyses were performed based on partial 18S rDNA gene and ITS region of the newly obtained sequences and selected sequences of the representatives of the superfamily Heterakoidea and genus *Heterakis* available in GenBank. The sequences were aligned using ClustalW multiple alignment implemented in Mega7 [18]. Both alignments were trimmed to the length of the shortest sequence. Phylogenetic analyses were conducted using Bayesian inference (BI) as implemented in MrBayes ver. 3.2.6 software [19]. The general time reversible model with estimates of invariant sites and gamma distributed among-site variation (GTR + I + G) was identified as the best-fitting nucleotide substitution model for both analyses using jModelTest 2 software [20]. Markov chain Monte Carlo (MCMC) chains were run for 2,000,000 generations, log-likelihood scores were plotted, and the final 75% of trees were used to produce the consensus trees. The trees were visualized in FigTree ver. 1.4.3 software [21].

## Results

### Necropsy Results and Parasites’ Identification

**Fig. 1:**
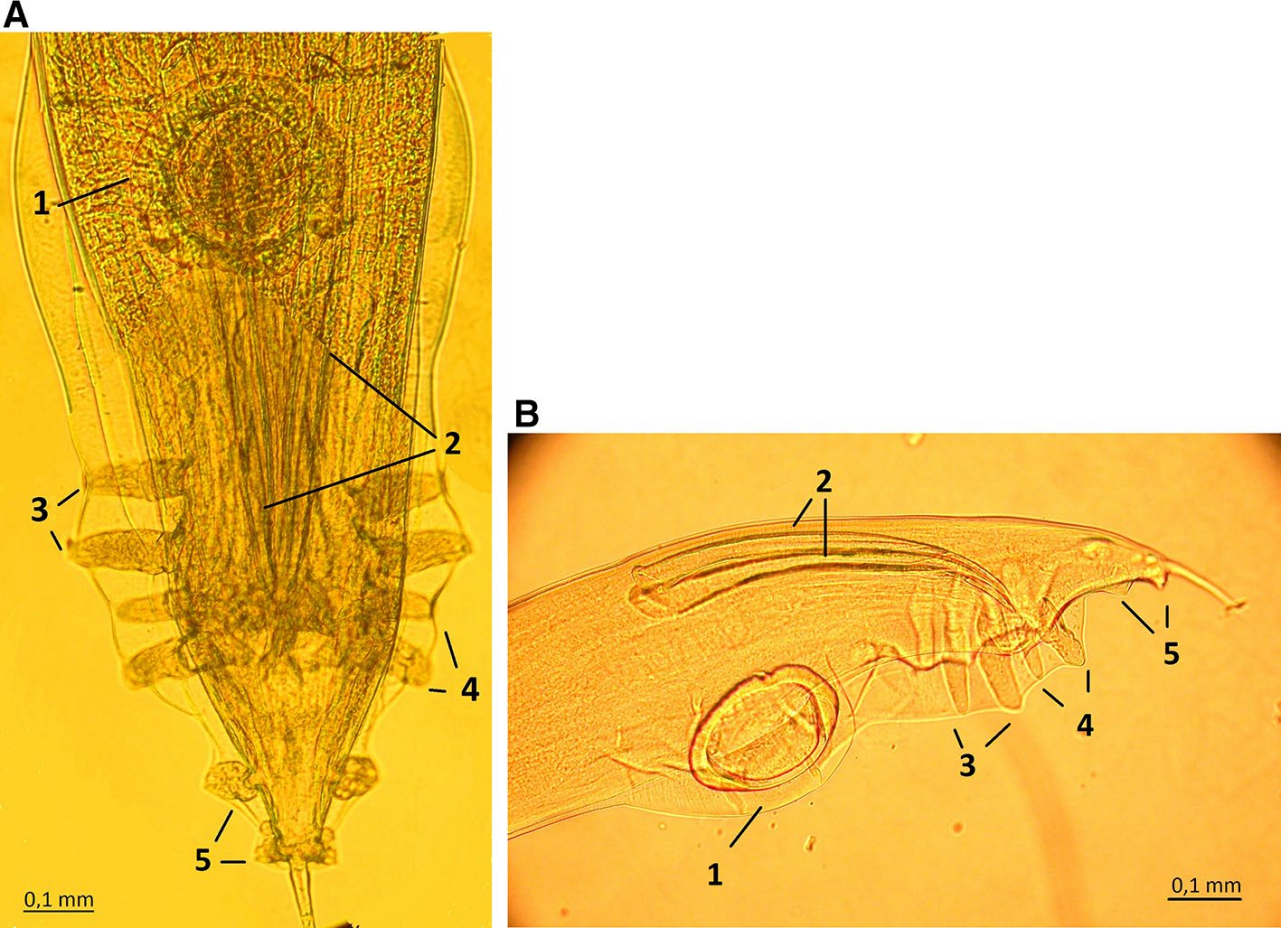
**a** Ventral side of tail end of *Heterakis dispar* male. **b** Lateral side of tail end of *Heterakis dispar* male. 1—Preanal sucker, 2—spicules, 3—preanal pairs of papillae, 4—adanal pairs of papillae, and 5—postanal papillae

The necropsied geese died because of peritonitis caused by *E. coli*. The nematodes were found in ceca, but no lesions of the cecal wall were observed. 15, 20, and 37 *Heterakis* specimens were obtained from three geese. Forty-five females and twenty-seven males were collected. All analyzed males isolated from the geese were characterized by short, equal-length spicule and 13 pairs of tail papillae—4 preanal, 4 adanal, and 5 postanal pairs what is typical for *Heterakis dispar* (Fig. 1a, b). The rest of morphometrical feature of males listed in the Table 1 also corresponded with the description of *H. dispar* provided by Madsen [5] and Yevstafyeva et al. [13].

**Table 1:** Morphometric characters of *Heterakis dispar* males

Characters (µm)	Data		References
	Size	*x* ± SD	Size or *x* ± SD
Length of the body	8175–11,625	9696.4 ± 946.4	1210–1480 [5] 10,000–15,000 [9] 13,360 ± 980 [13]
Width of the body at bulbus	320–350	328.6 ± 7.3	350–380 [9] 410 ± 30 [13]
Sucker diameter	180–205	195.0 ± 6.3	183–256 [5] 200 [9] 149.58 ± 5.03 [13]
Distance from preanal sucker to the tail end	480–635	545.7 ± 31.8	274–518 [5] 570 ± 60 [13]
Length of left spicule	400–460	434.3 ± 19.3	550–700 [5] 530–560 [9] 390 ± 20 [13]
Width of left spicule in the proximal end	26–35	29.1 ± 1.9	29.33 ± 1.01 [13]
Length of right spicule	410–410	437.1 ± 16.6	610–700 [5] 530–560 [9] 400 ± 10 [13]
Width of right spicule in the proximal end	26–30	28.8 ± 1.2	30.30 ± 1.34 [13]

### PCR Results and Molecular Analysis

**Fig. 2:**
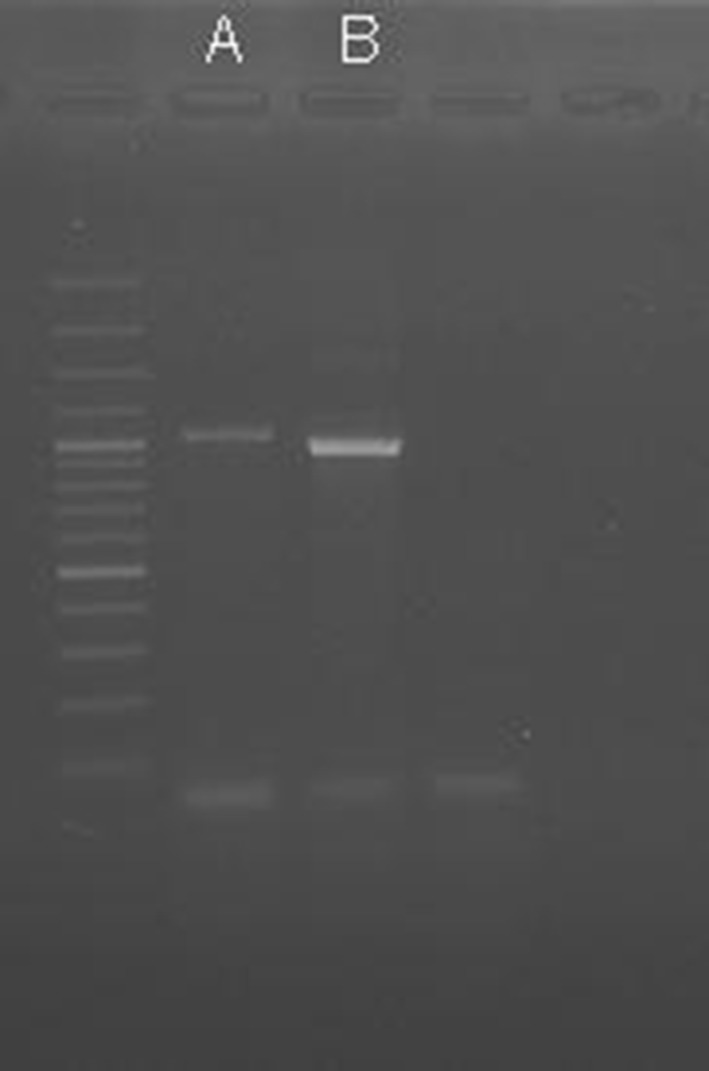
The PCR products of *H. gallinarum* (**a**) and *H. dispar* (**b**) with Thermo Scientific™GeneRuler™100 bp plus DNA ladder

The 18S rRNA PCR product of *H. dispar* was about 800 bp and was similar to the *H. gallinarum* product. The ITS-5.8S-ITS2 PCR product of *H. dispar* has a noticeably smaller size about 920 bp comparing to ~ 1100 bp *H. gallinarum* product (Fig. 2). The PCR reaction amplifying the ITS region turned out to be a good tool for differentiation *H. gallinarum* and *H. dispar*.

### The Phylogenetic Analysis

**Fig. 3:**
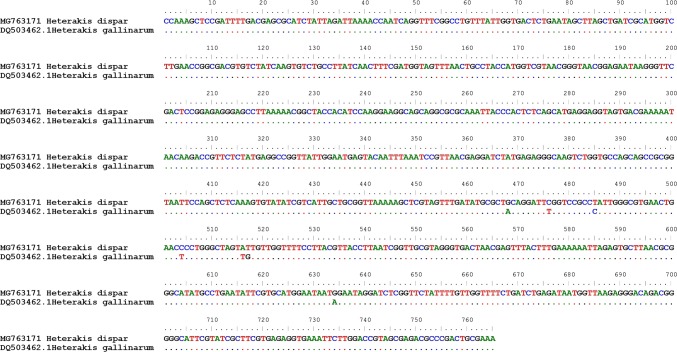
A comparison of 18S rRNA fragment of *H. dispar* and *H. gallinarum*

The provided comparative analysis of the newly obtained *H. dispar* sequences with the homological sequences available in the GenBank showed the molecular differentiation of representatives of the *Heterakis* genus recorded in geese. However, because of the lack of an 18S rRNA sequence of *H. isolonche* in the GenBank, we were able to compare only *H. dispar* with *H. gallinarum* on both genes. In the 18S rRNA sequence, seven substitutions—five transitions and two transversions—were observed. The substitutions were noted in the 468, 476, 485, 504, 516, 517, and 634 positions of the analyzed sequence (Fig. 3). The variety in the partial 18S rRNA gene between *H. dispar* and *H. gallinarum* is less than 1%.

**Fig. 4:**
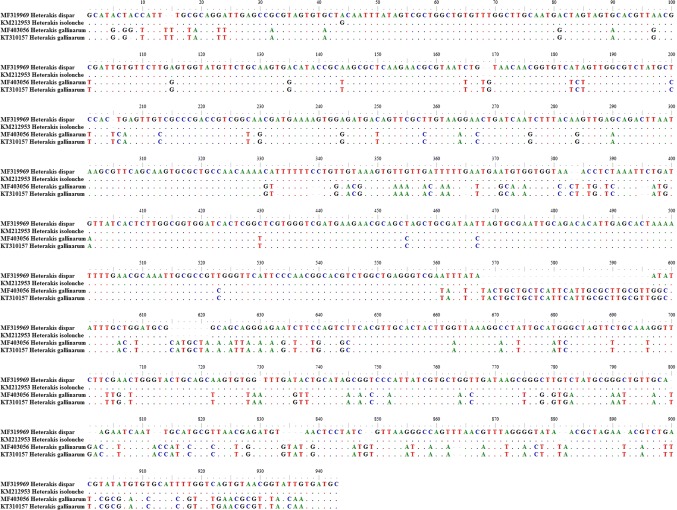
A comparison of ITS1-5.8S-ITS2 fragment of *H. dispar*, *H. gallinarum,* and *H. isolonche*

**Fig. 5:**
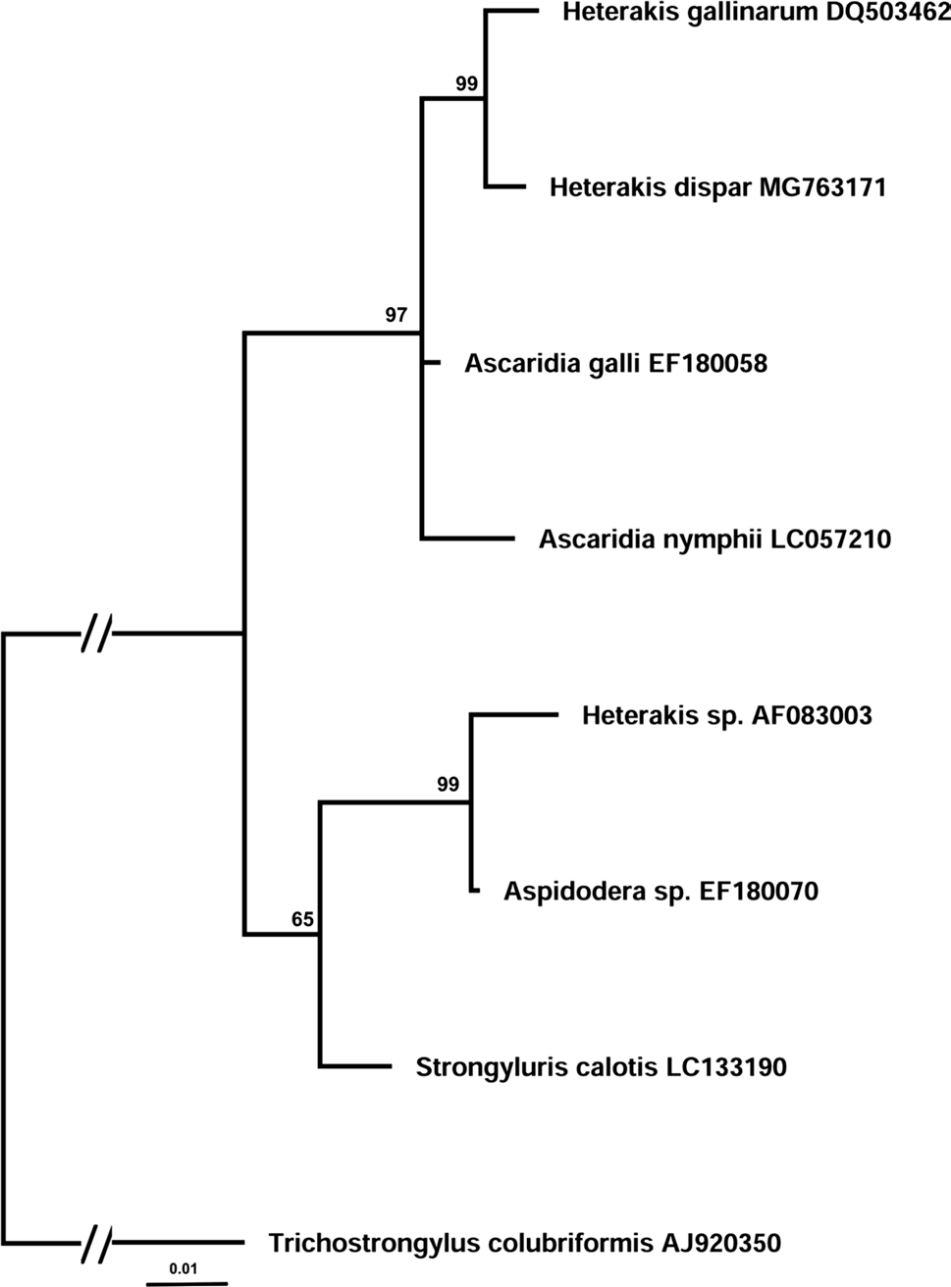
Phylogenetic interrelationships among representatives of Heterakoidea superfamily based on Bayesian analysis of partial sequences of the 18S rDNA gene. The scale bar indicates the number of substitutions per site. *Trichostrongylus colubriformis* was used as an outgroup

The comparison of the ITS region of the analyzed *H. dispar* to the H. *gallinarum* and *H. isolonche* sequences (943 bp) is shown in Fig. 5. The sequences of *H. beramparie* because of their shorter length were excluded from this analysis. In the conservative, protein coding 5.8S rRNA sequence, five substitutions in the 430, 455, 467, 524, and 561 positions, four transitions, and one transversion in the *H. dispar* sequence occurred according to the *H. gallinarum* sequence (Fig. 4). In the analyzed ITS1 region, there were 68 or 69 substitutions, and in the ITS2 region, 155 substitutions between *H. dispar* and *H. gallinarum* were noted. This yields a 24.2% variety in the ITS1-5.8S-ITS2 region sequence between those two species. Simultaneously, we have only noted one nucleotide substitution between the *H. dispar* and *H. isolonche*. This transversion in position 44 is in the ITS1 fragment.

The BLAST analysis of *H. dispar* 18S sequence (MG763171) showed a 99% similarity with the sequences of *H. gallinarum* (DQ503462) and *Ascaridia galli* (EF180058), 98% with *A. nymphii* (LC057210), but only 94% with the sequence of *Heterakis* sp. (AF083003). Our ITS sequence of *H. dispar* (MF319969) was almost identical to the *H. isolonche* isolate (KM212953); there is only one nucleotide of difference among the 943 sites analyzed. It also showed a lower similarity to the ITS sequences of *H. gallinarum* (88%, KT310157), *H. beramporia* (87%, KU529974.1), *H. spumosa* (87%, JX845278), and *H. dahomensis* (87%, JX845277). The differences between the ITS sequences of the *H. dispar* and *Ascaridia* species were larger. According to the BLAST analysis, *A. galli* KX683286 and *A. galli* KY789470 are similar to *H. dispar* in 80% (560 of the positions analyzed).

**Fig. 6:**
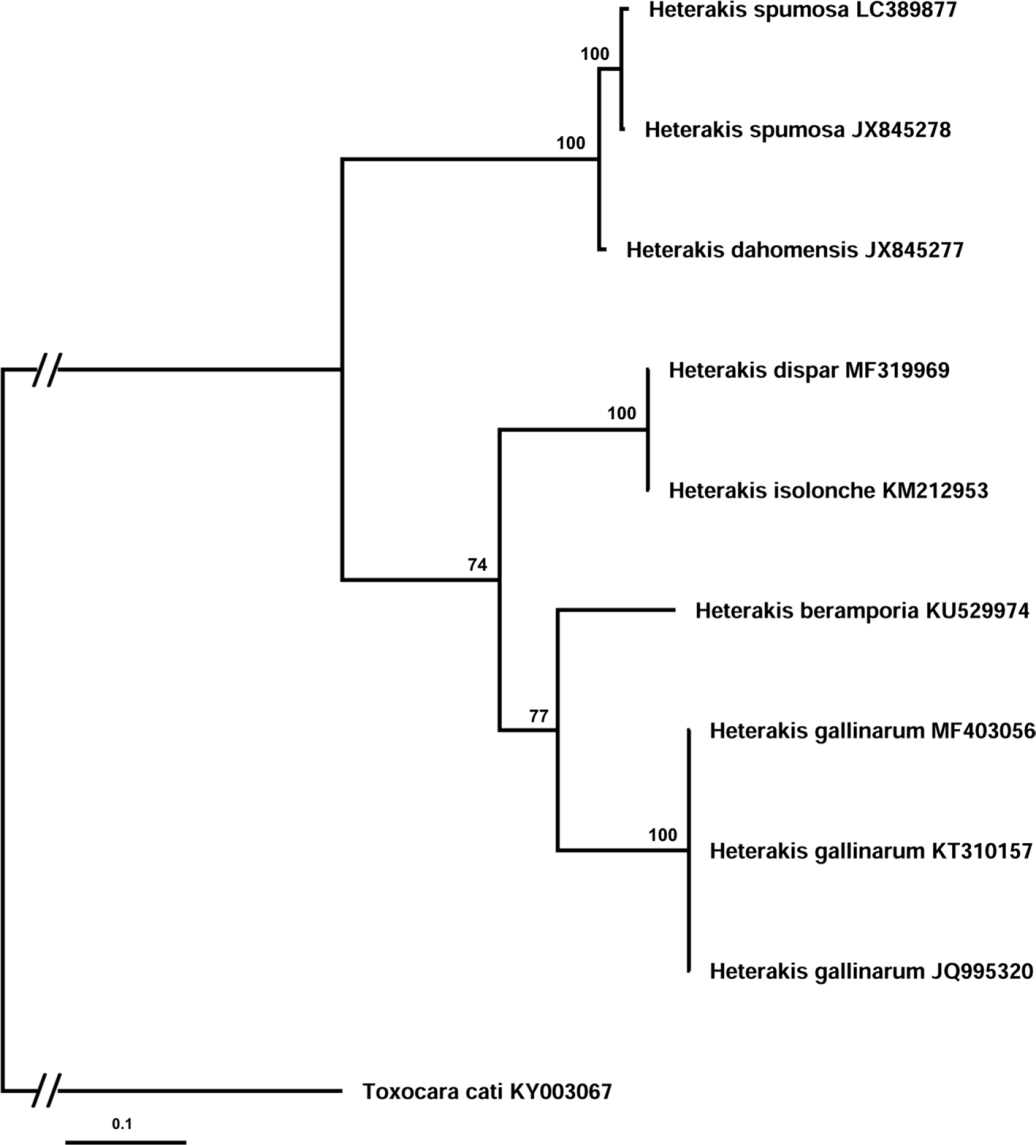
Bayesian analysis of ITS1-5.8S rDNA-ITS2 region sequences of *Heterakis* spp. constructed using MrBayes. The scale bar indicates the number of substitutions per site. *Toxocara cati* was used as an outgroup

In an attempt to create phylogenetic relationships of *H. dispar,* we performed the Bayesian inference (BI) analysis based on a partial 18S rRNA gene and ITS1-5.8S2-ITS2 region of representatives of *Heterakis* species and some related species from Heterakoidea. Unfortunately, our choice of molecular target was limited by sequences currently available in the GenBank. At least, the phylogenetic analysis involved 10 nucleotide sequences and a total of 1029 positions in the final dataset in ITS case, 8 sequences, and a total of 774 positions in the dataset in 18S case. The analysis of 18S rRNA generated a phylogenetic tree with topologies supported by differential (65–99) values. The tree revealed well-supported clade of bird’s parasites, i.e., *Heterakis* sp. and *Ascaridia* sp., with 97% branch support; however, not all members of *Heterakis* were clustered in the same group, but together with other representatives of superfamily, i.e., *Aspidodera* sp. and *Strongyluris* sp. (Fig. 5). The phylogenetic tree generated based on ITS region formed two clearly separated clades: one included the *Heterakis* species from birds (74% branch support) and the second well-supported (100%) contained mammalian heterakids (Fig. 6).

## Discussion

The morphology and ecology of the parasite have traditionally provided the basis for nematode taxonomy. Recent analyses of the rRNA gene sequences have allowed for a revision of nematode phylogeny and taxonomy [16, 22–24]. A group of genes, which encode three subunit rRNA (18S, 5.8S, and 28S) with external and internal sequences separating (ITS and ETS), are the most frequently used area for research. The differences in morphology between *H. gallinarum*, *H. isolonche*, and *H. dispar* noticed under the microscope [5, 9] showed that they are different species. Veterinarians who treat poultry flocks have no need to classify the cecal nematodes, because the antihelminth treatment does not depend on the *Heterakis* species. The flock treatment recommendations are made during the birds’ necropsy, and future analysis are not necessary for the owner. In our opinion, it might be one of the reasons, why there are not many sequences of nematodes isolated from poultry, including *Heterakis* spp. It is the first molecular analysis of *H. dispar* according to other nematodes. In our study, the structures typical for *H. dispar* were observed and the genetic analysis of the 18S rRNA gene and ITS1-5.8S-ITS2 region confirmed that this species differ from *H. gallinarum*. Ribas et al. [25] compared two mammal* Heterakis*—*H. spumosa* and* H. dahomensis*—define those species with an avarage of estimate of evolutionary divergence of 3.12 ± 0.83 base of differences per site. In our study, the analyzed ITS1-5.8S-ITS2 *H. dispar* sequence varied 25.5% from the *H. gallinarum*. It is difficult to relate to data of *H. isolonche*, because there is no 18S rRNA sequence and there is only one ITS1-5.8S-ITS2 sequence of this species, and no publication that could provide more details about this isolate. However, considering our and Ribas’ molecular results and with reference to the morphological data of *Heterakis* species, it was to be expected that differences between *H. dispar* and *H. isolonche* will be significant. In our opinion, the high similarity of ITS sequences of *H. dispar* and *H. isolonche* could suggest that there has been a mistake in the identification of the specimen from the China Rhine goose (GenBank accession number KM212953).

Our phylogenetic analysis is the first attempt at the reconstruction of relationships within this superfamily Heterakoidea, but has not been completed, because is still limited to a few representatives of this group of nematodes and fragmentary molecular data. Thus, our study complements the analysis provided by Nadler et al. [26].

The phylogenetic tree based on the ITS1-5.8S-ITS2 rRNA region shows a close relationship between heterakids parasitizing poultry, which are grouped into separated clade, and between nematodes isolated from rodents forming second clade. This result does not confirm former taxonomy of *Heterakis* based on morphometric features, e.g., this one was proposed by Skrjabin [27], who divided this genus according to the length of its spicules (equal vs. unequal) into *Ganguleterakis* and *Heterakis*.

## Conclusion

In this study, we analyzed the *Heterakis dispar* sequences, and provide a reconstruction of the relationships within the Heterakoidea family. The phylogenetic tree based on ITS1-5.8S-ITS2 rRNA sequences confirms a close relationship between poultry heterakids, which forms a separated clade on it. Further investigation looking at wider array of heterakids samples may shed light on the diversity within the genus and relationships within Heterakoidea superfamily.

## Abbreviation

ITS: Internal transcribed spacers
